# Effect of heat treatment on microbiological safety of supermarket food waste as substrate for black soldier fly larvae (*Hermetia illucens*)

**DOI:** 10.1016/j.wasman.2023.04.018

**Published:** 2023-06-01

**Authors:** Noor Van Looveren, Lotte Verbaet, Lotte Frooninckx, Sabine Van Miert, Leen Van Campenhout, Mik Van Der Borght, Dries Vandeweyer

**Affiliations:** a KU Leuven, Geel Campus, Department of Microbial and Molecular Systems (M^2^S), Research Group for Insect Production and Processing, Kleinhoefstraat 4, 2440 Geel, Belgium; b Thomas More University of Applied Sciences, RADIUS, Kleinhoefstraat 4, 2440 Geel, Belgium

**Keywords:** Food waste, Substrate, Thermal treatment, Black soldier fly larvae, *Hermetia illucens*, Foodborne pathogens

## Abstract

Supermarket food waste, constituting 13% of global food waste, can be upcycled as substrate for black soldier fly larvae (BSFL) and converted into larval biomass. Since presence of food pathogens in supermarket food waste is likely, microbiological safety should be ensured when using waste as insect substrate. Heat treatment provides a suitable pre-treatment to reduce microbiological contaminations. This study investigated the effect of different temperature–time combinations on the microbiological safety of supermarket food waste as BSFL substrate. Artificial supermarket food waste without meat and fish (SFW) was inoculated with both *Salmonella* and *Staphylococcus aureus* (7.0 log cfu/g) and treated at 50 and 60 °C for 10, 20 and 30 min. While 50 °C was insufficient for adequate pathogen reduction, 60 °C only required 10 min to reduce the Enterobacteriaceae and *S. aureus* counts to < 1.0 log cfu/g and for absence of *Salmonella* in 25 g. Heat-treated SFW could be stored for two days at ambient temperature or refrigerated without pathogen growth. Treatment of supermarket food waste containing meat and fish at 60 °C for 10 min caused similar results as for SFW, but *S. aureus* persisted (2.4 log cfu/g), possibly by protective effects of fat and/or proteins. Finally, BSFL rearing experiments on SFW revealed significantly higher larval mass, bioconversion efficiency and waste reduction than on Gainesville diet, with no notable differences between untreated and heat-treated SFW. Rearing BSFL on supermarket food waste is possible, and unsafe food waste can be heated to obtain safety without eliminating nutrients necessary for rearing.

## Highlights

• Supermarket food waste is suitable substrate for rearing black soldier fly larvae.
• High waste reduction of supermarket food waste by rearing black soldier fly larvae.
• Heat treatment of supermarket food waste is necessary for microbiological safety.
• Substrate composition influences the effect of heat treatment on food pathogens.
• Heat treatments applied on supermarket food waste did not influence larval growth.

## Graphical abstract

**Figure:**
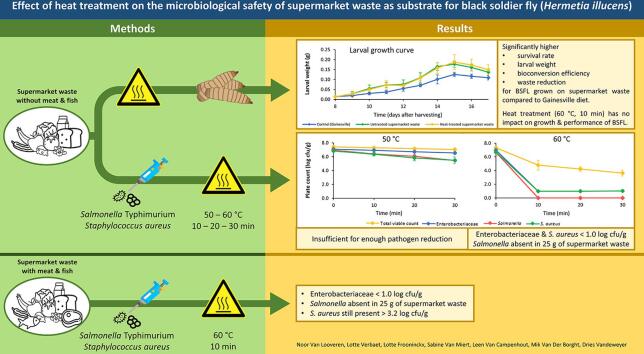

## 1 Introduction

Globally, about 931 million metric tons or 17% of the total food produced for human consumption is estimated to be wasted at the end of the human food supply chain (retail or consumption) each year. Supermarkets (or retail in general) are located almost at the end of this food supply chain and are a considerable source of food waste, since they represent 13% of the global food waste (United Nations, 2021). The largest wasted mass of food in supermarkets is often attributed to bread and fruit and vegetables, due to their short shelf life (Eriksson et al., 2016).

In addition to ethical, social and economic concerns, the environmental impact of food waste is also a major global challenge (Fao, 2013, Scholz et al., 2015). Hence, reuse of food waste would be a large step to a more sustainable world (Cicatiello et al., 2016, Papargyropoulou et al., 2014). This improvement would contribute to the UN Sustainable Development Goal on food loss and waste (SDG 12), in which is committed to halve global food waste per capita at retail and consumer level by 2030 (United Nations, 2016, United Nations, 2021). Reuse of food waste would also fit into the strategy of the three Rs of waste management (reduce, reuse, recycle) (Sakai et al., 2011) and it is in line with the European Directive 2008/98/EC on waste management (European Union, 2008).

One of the most promising valorization opportunities of food waste is providing it as substrate for insect rearing, such as the black soldier fly (*Hermetia illucens* L.) (Ojha et al., 2020). Black soldier fly larvae (BSFL) are able to grow on a wide range of organic materials, including food by-products, municipal waste and fruit and vegetable waste. They can convert these low-value waste streams into high-value biomass suitable for food, feed or other purposes. In this way BSFL play an important role towards a circular bioeconomy (Barbi et al., 2020, Magee et al., 2021, Siddiqui et al., 2022, Surendra et al., 2020).

Since insects are considered as farm animals, European Regulation (EC) No. 1069/2009 prohibits the use of manure, catering waste and former foodstuffs containing meat or fish as feed. Substrates that are allowed for insect production include commercially available animal feed and former foodstuffs that do not contain meat or fish, such as production excess or unsold foodstuffs with expired best-before-date (EFSA Scientific Committee, 2015; European European Commission, 2009, European Parliament and Council, 2002, IPIFF, 2020). Consequently, supermarket food waste, often called ‘swill’, is permitted as insect substrate, but only if it does not contain meat or fish.

When administering food waste to insects, rearers should be aware of its high microbial load, potentially carrying pathogenic microorganisms that can be harmful for animals or humans. Bacterial food pathogens such as *Staphylococcus aureus* and some pathogenic sulfite-reducing clostridia, were recently associated with insects produced for feed and food purposes and have already been detected in food waste obtained from supermarkets (Gorrens et al., 2021, Sancho et al., 2004). Rearing insects on contaminated substrates could potentially introduce these pathogens into the feed or food supply chain by potential accumulation and growth in the larvae. This effect is depending on the substrate and/or insect species and/or bacterial species (De Smet et al., 2021, Vandeweyer et al., 2021). Despite the possible microbiological risks, currently no microbiological criteria are set in the EU for insect feed. Only for the use of feed materials from animal origin, EU Regulation (EC) No. 142/2011 lays down microbiological standards for *Salmonella* and Enterobacteriaceae (European Commission, 2011).

To ensure microbiologically safe substrates for insects, a heat treatment can provide a suitable pre-treatment for food waste (Sancho et al., 2004). This is also suggested by the International Platform of Insects for Food and Feed (IPIFF) to reduce microbiological contaminations in former foodstuffs, also those containing meat and fish (IPIFF, 2020). Indeed, heat treatments, such as blanching, pasteurization and sterilization processes, are frequently used in food or feed production and mainly aim to reduce pathogenic or spoilage microorganisms (Ramaswamy and Marcotte, 2006). A large amount of heat treatment experiments evaluating the survival of microorganisms have already been performed. These studies demonstrated that the heat resistance of microorganisms depends on several factors, of which the treated matrix is one of the most important (Doyle et al., 2001, Fellows, 2016, Sancho et al., 2004). The heat sensitivity of a microorganism is generally characterized by a decimal reduction time (D-value, time needed for a tenfold reduction of the microbial count at a given temperature) and a z-value (increase in temperature needed for a tenfold reduction of the D-value) (Ramaswamy and Marcotte, 2006). In addition, a heating step also entails financial costs and may lead to overprocessing and loss of nutrients if not carefully optimized for the specific purpose. For this reason, it is important to find the most cost-efficient temperature–time combination for each situation.

In this study, the effect of different temperature–time combinations on the microbiological safety of supermarket food waste as substrate for BSFL rearing was investigated. Artificial supermarket food waste was created and inoculated with both *Salmonella* and *S. aureus*. *Salmonella* was selected given the criteria of Regulation (EU) No. 142/2011, used as basis for evaluation of microbiological safety. *S. aureus* was selected after observing high counts (up to 7.0 log cfu/g) of this pathogen upon preliminary microbiological screening of industrial food waste (Gorrens et al., 2021). The appropriate temperature–time combination to result in a microbiologically safe feeding substrate based on supermarket food waste not containing meat and fish (SFW), was also applied on supermarket food waste containing meat and fish (SFW + MF) and microbiological safety was evaluated again. Even though SFW + MF is currently prohibited, the aim was to provide scientific evidence to possibly expand the range of authorized substrates for insect rearing in the future. Finally, the potential of untreated and heat-treated SFW to be used as substrate for BSFL rearing was evaluated based on the larval development characteristics and performance.

## 2 Material and methods

### 2.1 Experimental set-up of heat treatment experiments

**Fig. 1:**
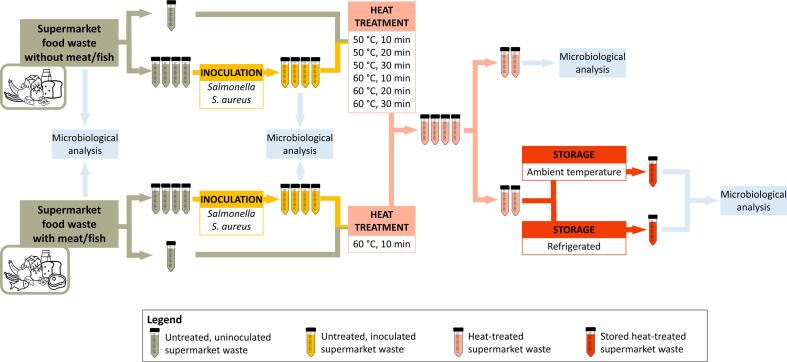
Experimental design of inoculation, heat treatment and storage of supermarket food waste not containing meat and fish (SW) and supermarket food waste containing meat and fish (SWMF). The experiment, including the preparation of the supermarket food waste, was performed in triplicate for both SW and SWMF.

The experimental set-up of the heat treatment experiments is illustrated in Fig. 1. SFW and SFW + MF were artificially created by mixing food ingredients, followed by inoculation with both *Salmonella* and *S. aureus*. Four tubes were filled with inoculated supermarket food waste, and an additional tube was filled with uninoculated supermarket food waste for temperature monitoring. Next, the tubes were heated in a water bath. For SFW, six combinations of temperature (50 and 60 °C) and time (10, 20 and 30 min) were applied. For SFW + MF, only the selected combination of 60 °C and 10 min was used. Microbial counts were determined before and after the heat treatment to evaluate the effect of the heat treatments. In addition to the two out of four heat-treated tubes (technical replicates) that were analyzed after the heat treatment, two other heat-treated tubes were stored for two days, one at ambient temperature and one in refrigerated conditions. After the storage, microbial counts were determined again. For both SFW and SFW + MF, three biological replicates were performed.

### 2.2 Supermarket food waste preparation

SFW was prepared based on the study of Lievens et al. (2022) and was composed of bread (22.0 wt%), lettuce (13.0 wt%), apple (12.0 wt%), courgette (12.0 wt%), cucumber (12.0 wt%), tomato (12.0 wt%), potato (9.0 wt%), kidney beans (6.0 wt%), cheese (1.5 wt%) and yoghurt (0.5 wt%). All ingredients were obtained from a local supermarket (Colruyt, Geel, Belgium) and stored maximally 24 h at 4 °C. Unwashed food ingredients were non-sterile blended with a home type kitchen mixer (Ergomixx, Bosch, Gerlingen, Germany). SFW + MF was prepared by adding 25.0 wt% of minced pork meat and 3.0 wt% of cod, obtained from the same supermarket, to the abovementioned artificial SFW, followed by blending with a home type kitchen mixer (Ergomixx, Bosch). As the nutritional values of the constituent ingredients are very well known and can be found in reliable databases, such as the Dutch Food Composition Database from the National Institute for Public Health and the Environment (Dutch Ministry of Health, Welfare and Sport) which is used in this study (RIVM, 2022), the nutritional composition of SFW and SFW + MF was calculated and is shown in Table 1. Further characterization of SFW and SFW + MF was performed by analysis of intrinsic parameters and microbial counts.

**Table 1:** Nutritional composition (protein, fat, carbohydrate, fiber and moisture content) of supermarket food waste not containing meat and fish (SFW) and supermarket food waste containing meat and fish (SFW + MF), based on nutritional values of the constituent ingredients (RIVM, 2022).

	**SFW**	**SFW + MF**
Protein (%)	3.5	8.0
Fat (%)	1.0	3.4
Carbohydrate (%)	15.3	11.1
Fiber (%)	1.8	1.3
Moisture (%)	77.8	74.7

### 2.3 Analysis of intrinsic parameters

Freshly prepared SFW and SFW + MF were subjected to measurement of pH, water activity (a_w_) and moisture content. A digital pH meter (Portamess 911, Knick, Berlin, Germany, with SI analytics electrode, Mainz, Germany) was used at ambient temperature for pH measurement. Water activity was determined using an a_w_-meter (LabMaster a_w_, Novasina, Lachen, Switzerland), after a_w_ and temperature (25 °C) were stable for 5 min. Moisture content was evaluated after oven drying of 5 g supermarket food waste at 105 °C for 17 h and calculating the weight loss. For each replicate of supermarket food waste prepared, pH, a_w_ and moisture content measurements were performed in triplicate (technical replicates).

### 2.4 Bacterial strains and inoculation

*Salmonella enterica* subsp. *enterica* serovar Typhimurium (LMG 18732) was obtained from the Belgian Coordinated Collection of Microorganisms (BCCM, Ghent, Belgium). To avoid difficulties in the detection of *Salmonella* by overgrowth of background bacteria (observed in preliminary experiments, data not shown), a kanamycin-resistant *Salmonella* Typhimurium strain was created as described by De Smet et al. (2021). The *Staphylococcus aureus* strain (LMG 8046) used was also obtained from the BCCM. Both bacterial strains were cultivated overnight in Luria-Bertani broth (LB, 10 g/L tryptone (VWR International, Leuven, Belgium), 5 g/L yeast extract (VWR International), 10 g/L NaCl) at 37 °C on a shaking plate (Orbital mini shaker, VWR International) at 150 rpm. For the kanamycin-resistant *Salmonella*, kanamycin (50 µg/mL, Fischer Scientific, Merelbeke, Belgium) was added to the LB.

Individual cultures were diluted with peptone physiological salt solution (0.1% peptone (Biokar Diagnostics, Beauvais, France), 0.85% NaCl) to a density of 4.0 McFarland units (MFu, measured with a DEN-1 McFarland 166 Densitometer, Grant instruments, Cambridge, UK), which corresponded with approximately 9.0 log cfu/mL for both bacterial strains (preliminary experiments, data not shown). To simulate the supermarket food waste to be highly contaminated, 5 mL of both *Salmonella* and *S. aureus* inoculation suspension were added drop by drop to 500 g of SFW or SFW + MF to reach an initial concentration of approximately 7.0 log cfu/g for both inoculants. The supermarket food waste was then homogenized with a sterile spoon. Finally, sterile cylindrical 50 mL tubes (2.8 cm diameter × 11.4 cm, polypropylene) were filled with aliquots of 40 g of the inoculated supermarket food waste for the heat treatment.

### 2.5 Heat treatment and storage of supermarket food waste

For SFW, six combinations of temperature (50 and 60 °C) and time (10, 20 and 30 min) were applied on the tubes filled with inoculated SFW. The tubes were submerged in a temperature-controlled water bath (WNB14, Memmert, Schwabach, Germany) and were treated for 10, 20 or 30 min after reaching the treatment temperature (50 or 60 °C) in the coldest point of the tube (determined in preliminary experiments, no data shown). An additional tube with uninoculated SFW was used to register the temperature profile using a temperature data logger (Escort Junior High Temperature logger HJ-FP-V-16-CI, Escort Messtechnik AG, Aesch, Switzerland). After the heat treatment, the tubes were placed in an ice bath for 30 min to cool down before analysis. Additionally, a heat treatment on SFW + MF was performed similarly, but only the selected temperature–time combination of 60 °C for 10 min was applied. For both SFW and SFW + MF, microbiological analyses were performed before and after the heat treatment. In addition, two heat-treated samples of each temperature–time combination were stored for two days, one at ambient temperature and one refrigerated (± 4 °C). Microbial counts were determined again after two days of storage.

### 2.6 Microbiological analyses

All microbial counts were determined according to the ISO-standards for microbial analyses of food and feed, as compiled by Dijk et al. (2015), except for the media used for *Salmonella* and *S. aureus* counts. For each sample, 5 g of supermarket food waste were diluted in 45 g of sterile peptone physiological salt solution to obtain a primary dilution, followed by homogenization for 1 min in a stomacher (BagMixer, Interscience, Saint Nom, France). A tenfold dilution series was prepared from this primary dilution and plated on different media. Total viable counts were determined on Plate Count Agar (PCA, Biokar Diagnostics) after incubation at 30 °C for 72 h. Enterobacteriaceae were determined on Violet Red Bile Glucose agar (VRBG, Biokar Diagnostics) after incubation at 37 °C for 24 h. The pathogen *S. aureus* was counted on Vogel-Johnson Agar spread plates (VJA, Merck Life Science, Hoeilaart, Belgium), supplemented with a 1% potassium tellurite solution (20 mL/L VJA, Merck Life Science), after incubation at 37 °C for 24 h. *Salmonella* spp. was counted on chromogenic RAPID’*Salmonella* agar spread plates (Bio-Rad Laboratories, Temse, Belgium), supplemented with kanamycin (50 µg/mL) and incubated at 37 °C for 48 h. After the heat treatment and after storage, presence of *Salmonella* in 25 g of supermarket food waste was determined using the RAPID’*Salmonella* short protocol (Bio-Rad Laboratories), according to the ISO 16140 standard. In short, selective enrichment was performed by diluting 25 g of heat-treated supermarket food waste in 225 mL of buffered peptone water (VWR International), with addition of a RAPID’*Salmonella* capsule (Bio-Rad Laboratories) for selective enrichment, and incubating at 41.5 °C for 18–22 h. Then, 10 µL of the enriched solution was spread on a RAPID’*Salmonella* plate, supplemented with kanamycin (50 µg/mL), and incubated at 37 °C for 24 h. A *Salmonella* latex kit (Bio-Rad Laboratories) was used to confirm presumptive *Salmonella* colonies. For each temperature–time combination, in total six replicates were analyzed, and plating was performed in duplicate to calculate the mean and standard deviation, expressed in log cfu/g.

After plotting the microbial counts of *Salmonella* and *S. aureus* in function of the treatment time, D-values could be calculated from the reciprocal of the slope of the linear trendline.

### 2.7 Black soldier fly rearing experiment

BSFL were obtained from a colony maintained by RADIUS (Thomas More University of Applied Sciences, Geel, Belgium). At day 0, 1 g of eggs was transferred to a small tray on top of 200 g of the nursery substrate, consisting of chicken starter feed (Chicken Start Mash 259, AVEVE, Belgium) and tap water in a 1:1 ratio (w/w). The container was incubated in a climate room at 27 °C and 60% relative humidity (RH). At day 4, the egg tray was removed, and the nursery substrate with small larvae was added to another box (29 × 19.5 × 18 cm), filled with 600 g substrate, being chicken starter feed and tap water in a 2:3 ratio (w/w), and incubated at 27 °C and 60% RH until day 8. For each rearing experiment, 500 8-day-old larvae were added to a plastic container (17.5 × 11.9 × 7.2 cm) filled with 500 g of one of the three different substrates. The substrates included artificial SFW (as prepared in ‘Supermarket food waste preparation’), artificial SFW heat-treated at 60 °C for 10 min, and Gainesville diet (50% wheat bran, 30% alfalfa, 20% corn meal, mixed with water to obtain 20% dry matter, similar to the dry matter of SFW) as control. The containers were covered with a lid with 2 holes (3 × 3 cm) to allow air circulation covered with a mosquito mesh to prevent larval escape. All containers were placed in a climate chamber (WEISS Pharma 600, Weiss Technik, Belgium) at 27 °C and 60% RH. For all three substrates, the rearing experiment was performed in triplicate (three biological replications), each time with two technical replications.

Larval weight was determined by daily weighing of 20 larvae from each container to set up a growth curve. At the end of the rearing experiment (day 17), larvae were harvested by first separating them from the substrate by sieving, followed by picking up the larvae with forceps. The larvae were counted and total larval end weight and dry matter content were determined. Total weight and dry matter content of the residue were also determined.

Larval survival rate was calculated by comparing the counted larvae before and after the rearing experiment. Bioconversion efficiency, bioconversion efficiency corrected for residue and waste reduction were calculated by Eqs. (1), (2), (3) (Bosch et al., 2020):

(1) $$\text{Bioconversion efficiency}\;\left(\%\right)\;=\;\left(L_{\mathrm{end}}-L_{\mathrm{start}}\right)/D\times 100\%$$

(2) $$\text{Bioconversion efficiency corrected for residue}\;\left(\%\right)\;=\;\left(L_{\mathrm{end}}-L_{\mathrm{start}}\right)/\left(D-R\right)\times 100\%$$

(3) $$\text{Waste reduction}\;\left(\%\right)\;=\;\left(D-R\right)/D\times 100\%$$

with *L_start_* and *L_end_* the total larval biomass at the start and end of the rearing experiment, respectively, *D* the amount of diet administered to the larvae and *R* the residue weight at the end of the rearing experiment. All parameters are expressed in g dry matter.

### 2.8 Statistical analyses

Intrinsic parameters and microbial counts of SFW and SFW + MF were statistically compared using an independent-samples t-test in case of normal distribution (evaluated by Shapiro-Wilk test) and equal variances (evaluated by Brown-Forsythe test). In case data were not normally distributed, a non-parametric Mann-Whitney U test was used. Statistical differences between plate counts of SFW after heat treatment and after storage were determined using one-way analysis of variance (ANOVA), followed by a Tukey HSD post-hoc test in case normality and homoscedasticity were confirmed. If data were not normally distributed, a non-parametric Kruskal-Wallis analysis was performed, followed by a Wilcoxon each pair test. The same statistical tests were used to compare plate counts before and after heat treatment and after storage of SFW + MF. Also for statistical comparison of the larval growth and process efficiency characteristics of the rearing experiment, ANOVA with Tukey HSD post-hoc test was performed in case of normal distribution and equal variances. In case normal distribution was not confirmed, a non-parametric Kruskal-Wallis test with a Wilcoxon each pair test was used. In case of unequal variances, Welch’s ANOVA with Steel-Dwass all pairs post-hoc test was used. For plate counts below the detection limit, the detection limit itself was used for statistical analysis. All statistical analyses were performed using the JMP Pro 16.0.0 software package from SAS. For each test, a level of significance of α = 0.05 was considered.

## 3 Results and discussion

### 3.1 Characterization of supermarket food waste

Both SFW and SFW + MF were characterized for intrinsic parameters and microbial counts. As intrinsic parameters, pH, a_w_ and moisture content were determined. Whereas SFW showed a pH of 4.17 ± 0.11, the addition of meat and fish resulted in a significantly higher pH of 4.82 ± 0.14 for SFW + MF (p < 0.001). While the pH of individual ingredients of the supermarket food waste was not determined in this study, other studies reported pH values of 5.6–5.9 for minced pork meat (Andritsos et al., 2012, Cauchie et al., 2020, Fengou et al., 2019). The higher pH of meat (and to a lesser extent of fish) than the SFW explains the pH increase in response to the addition of minced pork meat, which consisted 25 wt% in SFW + MF. In addition, high a_w_-values of 0.98 ± 0.01 and 0.97 ± 0.00 were recorded for SFW and SFW + MF, respectively, which are optimal for microbial growth (López-Malo and Alzamora, 2015). A moisture content of 78.2 ± 0.5% was obtained for SFW. This value was close to the calculated moisture content of 77.8% shown in Table 1 and to the moisture content of 82.8 ± 0.7% obtained for food waste from supermarkets and catering in a recent study (Broeckx et al., 2021). With the addition of meat and fish, the moisture content was reduced to 74.9 ± 0.7% (statistically significant; p < 0.001). This value approached the calculated moisture content of 74.7% for SFW + MF shown in Table1. Protein and fat content of SFW were calculated as 3.5% and 1.0%, respectively (Table 1), which are comparable to recently obtained actual values of 3.3% proteins and 1.9% fats by Broeckx et al. (2021). Addition of minced pork meat increased the protein and fat content of the food waste, illustrated by an increase to 8.0% protein and 3.4% fat for SFW + MF (Table 1). The fat content of a food matrix, and to a lesser extent also the protein content, is known to affect microbial inactivation during thermal treatments. Moreover, an increased fat content decreases the inactivation rate of a microorganism, resulting in an increase of its D-value. This protective effect is mainly attributed to cell entrapment leading to a lower heat conductivity and to reduction of the water activity of the matrix (Aguilar et al., 2013, Jay et al., 1992, Juneja and Eblen, 2000, Ramirez-Hernandez et al., 2018, Verheyen et al., 2021, Verheyen et al., 2020).

Unlike the intrinsic parameters, the microbial counts did not differ significantly between SFW and SFW + MF. For SFW, a total viable count of 6.5 ± 0.4 log cfu/g was observed and counts for the Enterobacteriaceae were 3.7 ± 0.6 log cfu/g. Similar counts were observed for SFW + MF and were 6.0 ± 0.5 log cfu/g for the total viable count and 4.1 ± 0.5 log cfu/g for the Enterobacteriaceae. For both SFW and SFW + MF, *Salmonella* was below the detection limit of 1.0 log cfu/g, while for coagulase-positive staphylococci, which is an indicator for the pathogen *S. aureus*, values of 3.3 ± 0.3 log cfu/g and 3.6 ± 0.2 log cfu/g were counted for SFW and SFW + MF, respectively. This amount of *S. aureus* cells does not necessarily pose a safety risk yet, since an infectious dose of 5.0 log cfu/g of *S. aureus* cells is generally considered as human threshold level for production of hazardous levels of enterotoxins (Hennekinne et al., 2012, Schmid-Hempel and Frank, 2007). As fresh food ingredients were used to artificially create supermarket food waste, microbial counts were lower compared to real food waste, for which counts for *S. aureus* up to 7.0 log cfu/g were previously reported (Gorrens et al., 2021, Sancho et al., 2004). Inoculation of *Salmonella* and *S. aureus* simulated the case of highly contaminated food waste.

### 3.2 Heat treatment of supermarket food waste not containing meat and fish

**Fig. 2:**
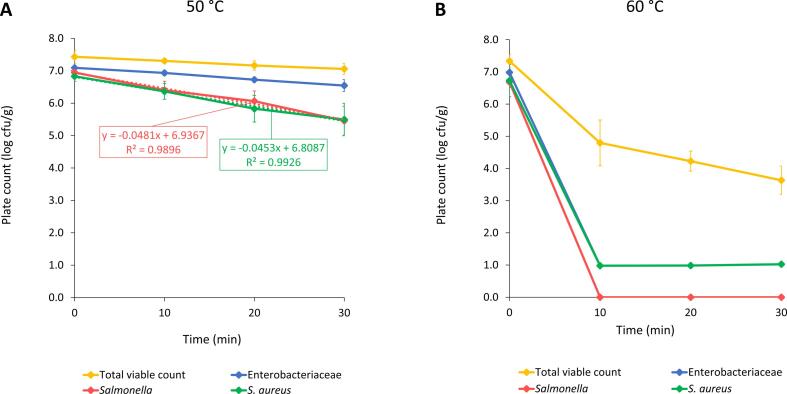
Reduction of microbial counts in supermarket food waste not containing meat and fish (SFW) after inoculation with *Salmonella* and *S. aureus* and after heat treatment of 10, 20 and 30 min at (A) 50 °C and (B) 60 °C. Results are presented as the mean of three experiments × two replicates per experiment (n = 6) ± standard deviation. For (A) 50 °C, a trendline with linear regression was represented by dashed lines with accompanying equation and R^2^-value for *Salmonella* and *S. aureus*.

Prior to the heat treatment, inoculation of SFW with *Salmonella* and *S. aureus* was performed to evaluate the effect of thermal treatment on the inactivation or survival of these pathogens in highly contaminated food waste. Fig. 2 shows the microbial counts of the SFW after inoculation and after 10, 20 and 30 min of heat treatment at 50 and 60 °C. Levels of *Salmonella* and *S. aureus* in the inoculated SFW were both 6.8 log cfu/g, which was close to the target inoculation level of 7.0 log cfu/g. The temperature profiles of the heat treatments are shown in Figure A1 (see Appendix).

As illustrated in Fig. 2, a treatment temperature of 50 °C only had limited impact on the microbial load. The total viable counts and Enterobacteriaceae were hardly reduced with less than one log-cycle and both pathogens were still present at 5.5 log cfu/g after 30 min of thermal treatment. These microbial counts pose a large safety risk, since the threshold levels for the infectious dose for *S. aureus* (5.0 log cfu/g) and *Salmonella* (between < 1.0 and 11.0 log cfu/g, depending on the serotype) were exceeded (Hennekinne et al., 2012, Schmid-Hempel and Frank, 2007, Teunis et al., 2010). The limited impact of a treatment temperature of 50 °C was also reflected in high D-values of the pathogens. These D-values could be calculated from the linear trendline for *Salmonella* and *S. aureus*, illustrated in Fig. 2. At 50 °C, the D-value was calculated as 20.8 min for *Salmonella* and 22.1 min for *S. aureus*. Theoretically, these D-values would lead to treatment times of at least 145.6 and 154.7 min to obtain a 7-log reduction for *Salmonella* and *S. aureus*, respectively. It should be noted that a D-value not only depends on the treatment time, but also on several other factors, such as growth phase of the bacterial culture, strain variations and composition of the surrounding matrix, such as pH, a_w_, and presence of proteins, fat or carbohydrates (Doyle et al., 2001, Fellows, 2016). Although a thermal treatment of 50 °C for 30 min only resulted in a small decrease of the added pathogens, a recent study of Gorrens et al. (2021) defined a reducing effect of BSFL on *S. aureus* in the rearing substrate and the larvae. Nevertheless, it is recommended eliminating *S. aureus* as soon as possible, since the pathogen can produce heat-stable enterotoxins (Hennekinne et al., 2012). In contrast, a reducing effect could not be observed for *Salmonella*. BSFL even ingested and accumulated this pathogen when present in the substrate. Consequently, it was recommended using substrates that do not contain *Salmonella* for BSFL rearing (De Smet et al., 2021). Based on these findings, it can be posed that a treatment temperature of 50 °C is insufficient to result in a microbiological safe substrate for BSFL within an acceptable timeframe. When taking into account the microbiological standards applying to animal by-products used in feed materials (Regulation (EU) No. 142/2011), which imposes *Salmonella* to be absent in 25 g of sample (European Commission, 2011), inadequacy of a thermal treatment of 50 °C for up to 30 min was confirmed.

A treatment temperature of 60 °C clearly had a larger reducing effect on all microbial counts (Fig. 2). A treatment time of 10 min resulted in a reduction of the total viable counts to 4.8 log cfu/g. Each additional 10 min of treatment caused an additional decrease of 0.6 log-cycles to reach an average total viable count of 3.6 log cfu/g after 30 min. A notable impact could be observed for the Enterobacteriaceae, which were reduced to below the detection limit of 1.0 log cfu/g after 10 min at 60 °C. Also, counts for both pathogens studied were below this detection limit, with *Salmonella* even absent in 25 g of SFW. Unsurprisingly, D-values of *Salmonella* and *S. aureus* at 60 °C were much lower compared to those at 50 °C and were calculated as <1.5 and <1.7 min, respectively. When considering the microbiological standards for animal by-products used in feed (Regulation (EU) No. 142/2011), a treatment of 60 °C for 10 min seemed to be sufficient to meet both criteria for the Enterobacteriaceae (<300 cfu/g) and *Salmonella* (absent in 25 g). Consequently, it can be posed that a treatment time of 10 min at a temperature of 60 °C can be a possible temperature–time combination to result in a microbiologically safe substrate for BSFL. This combination could also be used at larger scales, provided that 60 °C is measured for 10 min in the coldest point of the large heat-treated volume in industrial installations.

Nevertheless, some important remarks should be made. A first remark is the impact of the coming-up time (CUT) of the heat treatment, which is the time needed to reach the imposed treatment temperature in the coldest point of the sample. This CUT is likely to have a reducing effect on the microbial load. In this study, the CUT was measured to be 25 min for both 50 and 60 °C and is indicated in the temperature profile shown in Figure A1 (see Appendix). When upscaling thermal treatment of substrates to an industrial scale and/or using other heating equipment, variations in the CUT and its influence on the microbial counts should be considered. In larger scale installations, the CUT is expected to be extended, resulting in higher reduction of the microbial counts and potentially even in overprocessing. In addition to the CUT, upscaling to an industrial scale would also significantly affect other factors, such as the dimensions and heat transfer. These factors will influence the costs related to the heat treatment as additional unit operation. In order to perform accurate cost analyses on the heat treatment of SFW, it is critical to have more information on the upscaling of this operation.

A second remark is that, in addition to the abovementioned microbiological safety risks, also other food pathogens could possibly pose a safety risk. Particularly spore-forming bacteria, such as pathogenic *Clostridium* spp. and pathogenic species of the *Bacillus cereus* group, which are some of the most relevant pathogens associated with insects, are noteworthy (Vandeweyer et al., 2021). Bacterial endospores are likely to survive the thermal treatments used in this study, since they are more resistant to treatments that inactivate vegetative cells from the same strain. Endospores can withstand even 40 °C higher temperatures compared to the vegetative cells (Setlow, 2006, Vandeweyer et al., 2020). Moreover, the mild heat treatment can even activate the endospores and possibly result in germination, multiplication and/or toxin production (Vandeweyer et al., 2020). However, the low pH of the supermarket food waste might reduce the spore germination process (Vandeweyer et al., 2020, Wheeldon et al., 2008), but risk of germination increases by pH increase when adding meat and fish to SFW. An appropriate microbiological safety assessment is highly recommended for endospore control. A third remark is that heat resistance of microorganisms can vary between bacterial strains or serotypes of the same genus (Jarvis et al., 2016). Additionally, the kanamycin-resistant *Salmonella* strain used in this study might possibly be more heat sensitive than wild-type *Salmonella* strains (Wang et al., 2021). Hence, it might be appropriate to repeat the heat treatment experiments with other bacterial strains to confirm the results of this study.

### 3.3 Storage of heat-treated supermarket food waste not containing meat and fish

After the heat treatment, the SFW was stored for two days at ambient temperature as well as refrigerated. The microbial counts before and after the storage are given in Table2 for all temperature–time combinations. For SFW treated at 50 °C, cold storage resulted in a slight (but never statistically significant) decrease in all plate counts and for all preceding treatment times, while storage at ambient temperature led to an increase in the plate counts. The pathogens *Salmonella* and *S. aureus* even grew out to values comparable to those before the heat treatment (around 7.0 log cfu/g). Besides the fact that a treatment temperature of 50 °C was considered insufficient, storage at ambient temperature would deteriorate the microbiological safety of SFW.

**Table 2:** Microbial counts after heat treatment of supermarket food waste not containing meat and fish (SFW), after cold storage and storage at ambient temperature for two days. Results are presented as the mean of three experiments (n = 3) ± standard deviation.

**Treatment time**	**Sample**	**Microbial counts (log cfu/g)**			
		**50 °C**			
		**Total viable count**	**Enterobacteriaceae**	***Salmonella***	***Staphylococcus aureus***
10 min	After heat treatment	7.3 ± 0.1^a^	6.9 ± 0.1^a^	6.4 ± 0.3^a^	6.4 ± 0.2^a^
	After cold storage	7.2 ± 0.2^a,b^	6.8 ± 0.1^a^	5.9 ± 0.8^a^	5.9 ± 0.4^a^
	After storage at ambient temperature	7.9 ± 0.4^b^	7.6 ± 0.1^b^	6.9 ± 0.3^a^	6.8 ± 1.3^a^
20 min	After heat treatment	7.2 ± 0.1^a^	6.7 ± 0.1^a^	6.1 ± 0.3^a^	5.8 ± 0.4^a^
	After cold storage	7.1 ± 0.2^a^	6.6 ± 0.1^a^	5.4 ± 0.3^a^	5.4 ± 0.3^a^
	After storage at ambient temperature	7.7 ± 0.5^b^	7.6 ± 0.3^b^	7.4 ± 0.9^b^	6.3 ± 1.1^a^
30 min	After heat treatment	7.1 ± 0.2^a^	6.5 ± 0.2^a^	5.5 ± 0.4^a^	5.5 ± 0.5^a^
	After cold storage	7.0 ± 0.1^a^	6.1 ± 0.2^a^	5.2 ± 0.2^a^	5.0 ± 0.3^a^
	After storage at ambient temperature	7.4 ± 0.4^a^	7.1 ± 0.4^b^	6.9 ± 0.7^b^	6.2 ± 1.0^a^

**Treatment time**	**Sample**	**60 °C**			
		**Total viable count**	**Enterobacteriaceae**	***Salmonella***	***Staphylococcus aureus***^†^
10 min	After heat treatment	4.8 ± 0.7^a^	<1.0 ± 0.0^a^	Absent in 25 g	<1.0 ± 0.0^a^
	After cold storage	4.0 ± 0.5^a^	<1.0 ± 0.0^a^	Absent in 25 g	<1.3 ± 0.6^a^
	After storage at ambient temperature	5.8 ± 1.0^a^	<1.0 ± 0.1^a^	Absent in 25 g	<1.3 ± 0.5^a^
20 min	After heat treatment	4.2 ± 0.3 ^a,b^	<1.0 ± 0.0^a^	Absent in 25 g	<1.0 ± 0.0^a^
	After cold storage	3.4 ± 0.3^a^	<1.0 ± 0.0^a^	Absent in 25 g	<1.3 ± 0.6^a^
	After storage at ambient temperature	6.3 ± 2.3^b^	<1.0 ± 0.0^a^	Absent in 25 g	<1.3 ± 0.6^a^
30 min	After heat treatment	3.6 ± 0.4^a^	<1.0 ± 0.1^a^	Absent in 25 g	<1.0 ± 0.1^a^
	After cold storage	3.4 ± 0.3^a^	<1.0 ± 0.0^a^	Absent in 25 g	<1.3 ± 0.6^a^
	After storage at ambient temperature	6.0 ± 2.0^b^	<1.0 ± 0.0^a^	Absent in 25 g	<1.3 ± 0.6^a^

^a,b^Means of samples of the same treatment temperature and time with the same letter in superscript in the same column do not differ significantly (p ≥ 0.05).

^†^For SFW heat treated at 60 °C, the detection limit of *S. aureus* after storage was set at 1.0 log cfu/g for two replicates and at 2.0 log cfu/g for one replicate, resulting in an average detection limit of 1.3 log cfu/g.

For SFW treated at 60 °C, similar effects of storage were observed for the total viable counts. Cold storage resulted in a limited decrease in total viable counts, whereas storage at ambient temperature caused an increase in total viable counts of at least one log-cycle. Although the total viable counts of the stored SFW reached values of around 6.0 log cfu/g, this does not necessarily imply a problem, since the total viable count only gives a quantitative estimate of all living microorganisms in the SFW but does not give specific insight in the presence of specific harmful microorganisms. For the Enterobacteriaceae and *S. aureus*, which were below the detection limit of 1.0 log cfu/g after the heat treatment, both cold storage and storage at ambient temperature retained values below this detection limit. *Salmonella* also remained absent in 25 g of SFW after two days of storage. These observations imply that SFW heat-treated at 60 °C could still be considered as microbiologically safe substrate for BSFL after at least two days of refrigerated storage or storage at ambient temperature, based on the microbiological standards of Regulation (EU) No. 142/2011. This could be an interesting opportunity for insect rearers, since they would be able to preserve heat-treated SFW for (at least) two days instead of being obliged to use treated substrates immediately. Further research is needed to determine the maximal storage period of this substrates after the appropriate thermal treatment.

### 3.4 Heat treatment of supermarket food waste containing meat and fish (10 min at 60 °C)

After selecting the temperature–time combination of 60 °C and 10 min as most suitable to result in microbiologically safe SFW (within the combinations tested), this combination was also applied on SFW + MF inoculated with *Salmonella* and *S. aureus*. The resulting plate counts before and after the heat treatment are given in Table 3. Before the heat treatment, all plate counts were around 7.0 log cfu/g as result of the inoculation with *Salmonella* and *S. aureus*. After the treatment, average total viable counts were significantly reduced to 3.8 log cfu/g (p < 0.001). Similar to the results for SFW (Fig. 2), the Enterobacteriaceae were reduced to below the detection limit of 1.0 log cfu/g, and *Salmonella* was absent in 25 g of SFW + MF. Surprisingly, the results for *S. aureus* in SFW + MF did not correspond to those obtained in SFW, as the counts were 2.4 log cfu/g after the thermal treatment. A possible reason for the survival of *S. aureus* can be the protective effect of the surrounding matrix, which was enriched with fat (and proteins) by addition of minced pork meat and fish to the SFW (see also Table 1). Indeed, a higher fat content may allow entrapment of bacterial cells, which can protect them to heat stress during thermal processing (Aguilar et al., 2013, Ramirez-Hernandez et al., 2018). Although other studies also reported increased heat resistance of *Salmonella* due to a higher fat content of the surrounding matrix (Juneja and Eblen, 2000, Ramirez-Hernandez et al., 2018), the protecting effect of fat on *Salmonella* could not be demonstrated in this study.

**Table 3:** Microbial counts of supermarket food waste containing meat and fish (SFW + MF) before and after heat treatment (60 °C, 10 min) and after storage. Results are presented as the mean of three experiments × two replicates per experiment (n = 6) ± standard deviation.

	**Microbial counts (log cfu/g)**			
	**Total viable count**	**Enterobacteriaceae**	***Salmonella***	***Staphylococcus aureus***
Before heat treatment	7.3 ± 0.0^c^	6.9 ± 0.0^b^	6.4 ± 0.6^b^	7.1 ± 0.0^d^
After heat treatment	3.8 ± 0.3^a^	<1.0^a^	Absent in 25 g^a^	2.4 ± 0.1^b^
After cold storage	4.9 ± 0.3^b^	<1.0^a^	Absent in 25 g^a^	2.1 ± 0.1^a^
After storage at ambient temperature	7.0 ± 0.9^c^	<1.0^a^	Absent in 25 g^a^	>3.2 ± 0.0^c†^

^a,b,c,d^Mean values with the same letter in superscript in the same column do not differ significantly (p ≥ 0.05).

^†^Results are calculated as ‘estimated value’, since the maximal number of countable colonies was exceeded for some of the replicates.

Similar to heat-treated SFW, SFW + MF was also stored in cold conditions and at ambient temperature (Table 3). Storage always resulted in a statistically significant increase in total viable counts, and the increase was larger for storage at ambient temperature (p < 0.001) than for cold storage (p = 0.015). Again, the amount of Enterobacteriaceae was still below the detection limit of 1.0 log cfu/g after storage and *Salmonella* was still absent in 25 g of SFW + MF. The fact that *S. aureus* survived the heat treatment of 60 °C for 10 min also led to presence of this pathogen in the stored SFW + MF. While cold storage slightly reduced the *S. aureus* counts to 2.1 log cfu/g (p = 0.002), storage at ambient temperature significantly increased the counts to above 3.2 log cfu/g (p < 0.001). These counts are still below the infectious dose threshold level for *S. aureus* (5.0 log cfu/g), but further growth and exceeding this level should be highly prevented.

When considering the microbiological standards for animal by-products used in feed (Regulation (EU) No. 142/2011), a treatment of 60 °C for 10 min can be assessed to be sufficient for SFW + MF to meet the required criteria for the Enterobacteriaceae and *Salmonella*. However, this study demonstrated that the survival of *S. aureus* after a heat treatment of 60 °C for 10 min depended on the heated matrix. While this pathogen was reduced to below the detection limit of 1.0 log cfu/g in SFW, thermal treatment of SFW + MF still resulted in survival of *S. aureus*. This observation indicates the importance of the composition of the substrate related to heat treatment of insect substrates. The presence and numbers of *S. aureus*, but also of other important food pathogens, should be closely monitored when using heat-treated supermarket food waste as substrate for BSFL.

### 3.5 BSFL rearing experiments

**Fig. 3:**
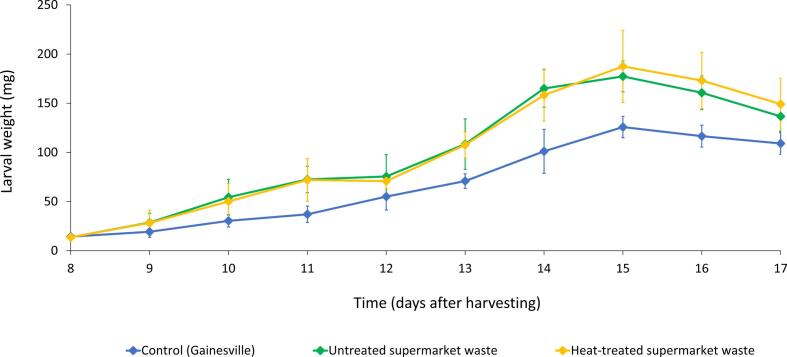
Larval growth curve of BSFL reared on a control diet (Gainesville diet), untreated supermarket food waste and heat-treated supermarket food waste for 10 days. Results are presented as the mean of three experiments × two replicates per experiment (n = 6) ± standard deviation.

Based on the larval weight during the rearing experiment, a growth curve (Fig. 3) was created for larvae grown on untreated SFW, heat-treated SFW and Gainesville diet (control). During the whole experiment, mean larval weight was higher for BSFL grown on SFW compared to the control. While the maximal larval weight was reached at day 15 for all substrates, it was significantly higher for larvae grown on untreated SFW (178.3 mg; p = 0.013) and heat-treated SFW (188.3 mg; p = 0.013) than for the control (126.7 mg), as also shown in Table 4. Comparable maximal larval weights were reported in the study of Broeckx et al. (2021), in which 400 g of twelve organic waste streams were fed to 500 BSFL during nine days. They reported a high average maximal larval weight of 176.4 mg for industrial food waste, which consisted of supermarket and restaurant food waste. Notably, no differences could be observed between the growth curve of untreated and heat-treated SFW (Fig. 3). This similarity demonstrates that the heat treatment of 60 °C for 10 min did not have a negative or positive effect on the growth characteristics of the BSFL. Consequently, it can be presumed that the heat treatment had no detrimental or promoting effect on the nutritional quality of the substrate and growth-promoting microorganisms for BSFL.

**Table 4:** Larval development and performance characteristics of BSFL reared on Gainesville diet (control), untreated supermarket food waste and supermarket food waste heat treated at 60 °C for 10 min. Results are presented as the mean of three experiments × two replicates per experiment (n = 6) ± standard deviation.

	**Control**	**Untreated SFW**	**Heat-treated SFW**
Survival rate (%)	87.0 ± 3.8^a^	98.5 ± 1.0^b^	98.6 ± 1.2^b^
Maximal larval weight (mg)	126.7 ± 12.1^a^	178.3 ± 16.0^b^	188.3 ± 38.7^b^
Bioconversion efficiency (%)	11.5 ± 1.5^a^	21.3 ± 2.5^b^	22.3 ± 3.4^b^
Bioconversion efficiency corrected for residue (%)	24.0 ± 1.6^a^	30.9 ± 4.8^b^	30.4 ± 3.2^b^
Waste reduction (%)	47.9 ± 3.6^a^	69.6 ± 8.9^b^	73.3 ± 7.0^b^
Residue dry matter (%)	53.3 ± 9.1^a^	77.4 ± 7.7^b^	68.0 ± 16.7^a,b^

^a,b^Mean values with the same letter in superscript in the same row do not differ significantly (p ≥ 0.05).

As shown in Table 4, more than 98% of the larvae grown on SFW survived, while average survival was significantly lower on Gainesville diet (87.0%; p < 0.001). Bioconversion efficiency was similar for untreated SFW (21.3%) and heat-treated SFW (22.3%), and was even double of the bioconversion efficiency on Gainesville diet (11.5%). A comparable bioconversion efficiency of 20.7% was already previously found for BSFL reared on industrial food waste (Broeckx et al., 2021). It can be stated that SFW (or food waste in general) can be efficiently converted into larval biomass. In future research, it may be worthwhile to characterize the nutritional composition of this larval biomass and compare it to the composition of the untreated and heat-treated SFW.

Since the feeding substrate or diet is mixed with larval excreta and (parts of) dead larvae during the rearing phase, the formula for bioconversion efficiency can be corrected for the residue left after the rearing experiment (often called ‘frass’). This corrected formula provides better insight in the relation between consumed substrate and biomass production (Bosch et al., 2020). The corrected bioconversion efficiency was still higher for untreated SFW (30.9%) and heat-treated SFW (30.4%) than for the control (24.0%), but the difference narrowed.

Furthermore, waste reduction was around 70% for untreated and heat-treated SFW, which is significantly higher compared to Gainesville diet (47.9%). Other studies reported waste reduction ranging from 45 to 70%, but highly depending on the substrate composition and associated nutrient content (Broeckx et al., 2021, Diener et al., 2011, Lalander et al., 2019, Lalander et al., 2015). The remaining residue can be used as fertilizer for plants or as soil improver, but a heat treatment of at least 70 °C for 60 min is required in the EU (Van Looveren et al., 2022). As the required amount of thermal energy depends on the moisture content of the frass, a higher dry matter content requires a lower drying energy (Kudra, 2004). The dry matter of the residue varied between the substrates, with the lowest value for the control substrate (53.3%) and higher values for heat-treated SFW (68.0%) and untreated SFW (77.4%). Since the average dry matter of the untreated and heat-treated SFW used as substrate was only 22.4% and 22.7%, respectively, rearing BSFL on SFW highly reduced the moisture content of the waste stream. From an energy point of view, it is more energy efficient to treat the dry residue instead of the wet substrate.

In general, supermarket food waste appears to be a nutritionally rich waste stream for rearing BSFL, with considerably high yields of larval biomass and substantial waste reduction. These promising results were observed for untreated SFW as well as for heat-treated (60 °C, 10 min) SFW. Consequently, reuse of supermarket food waste can be called ‘upcycling’. This means that low-value food waste, that would not have gone to human consumption otherwise, can be returned back into the feed or food production chain to result in value-added products (Lasaridi and Stentiford, 2011, Moshtaghian et al., 2021).

## 4 Conclusion

This study investigated the effect of different heat treatment temperature–time combinations on the microbiological safety of supermarket food waste as possible substrate for BSFL rearing. For SFW inoculated with 7.0 log cfu/g of *Salmonella* and *S. aureus*, a heat treatment at 50 °C was insufficient for microbiological safety. In contrast, a treatment at 60 °C only needed 10 min to reduce the Enterobacteriaceae and *S. aureus* to below the detection limit of 1.0 log cfu/g and to result in absence of *Salmonella* in 25 g of SFW. Storage of this heat-treated SFW in cold conditions and at ambient temperature turned out to be possible for (at least) two days without increasing the pathogen levels.

Next, the selected heat treatment (60 °C for 10 min) of SFW + MF resulted in similar results as for SFW, except for *S. aureus*, and can be considered sufficient for supermarket food waste to result in microbiological safety. Hence, this study demonstrated that the survival of *S. aureus* during heat treatment depends on the surrounding matrix. Consequently, presence of *S. aureus*, and also other important food pathogens, should be closely monitored.

Finally, rearing experiments with BSFL on SFW resulted in high larval mass, bioconversion efficiency and waste reduction compared to Gainesville diet. Additionally, heat treatment of SFW had no negative or positive effect on the growth characteristics and performance of the BSFL. It can be concluded that rearing BSFL can be a suitable solution for processing supermarket food waste, and an appropriate heat treatment can be used to obtain microbiologically safe waste without eliminating necessary nutrients for BSFL rearing.

## Declaration of Competing Interest

The authors declare the following financial interests/personal relationships which may be considered as potential competing interests: Noor Van Looveren reports financial support was provided by Research Foundation Flanders. Lotte Frooninckx reports financial support was provided by Agency of Innovation and Entrepreneurship. Dries Vandeweyer reports financial support was provided by EU Framework Programme for Research and Innovation Societal Challenges.

## Data Availability

Data will be made available on request.
