# Supplementary material for: Effect of heat treatment on microbiological safety of supermarket food waste as substrate for black soldier fly larvae (Hermetia illucens)
Source: Waste Manag. 2023 Jun 1;164:209–18. doi: 10.1016/j.wasman.2023.04.018 (PMC10162384; doi:10.1016/j.wasman.2023.04.018)
Supplement: Supplementary data 1 [file mmc1.docx]

**Appendix**


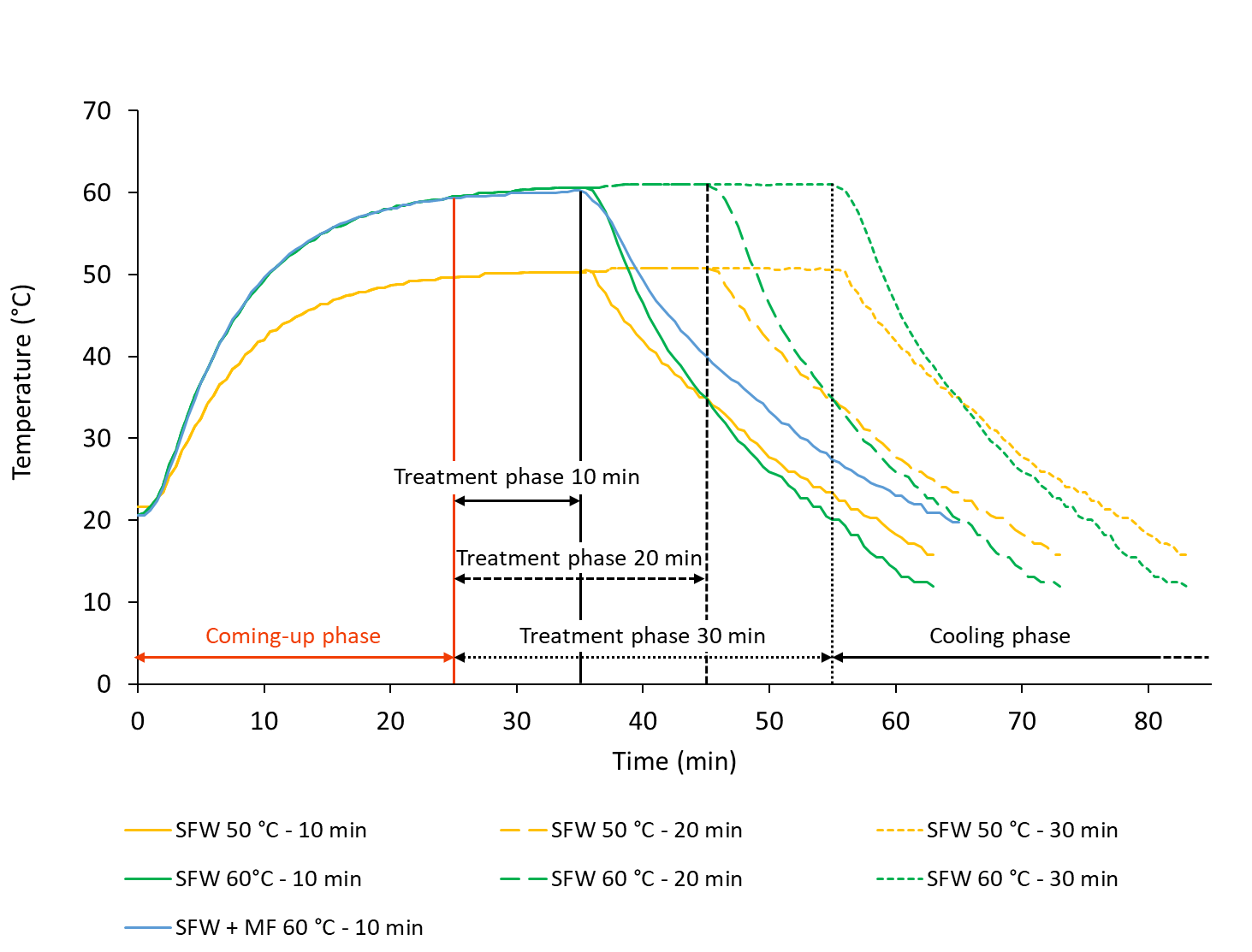


**Figure A1.** Temperature profiles (coming-up phase, treatment phase and cooling phase) of heat treatments (50, 60°C - 10, 20, 30 min) applied on supermarket food waste not containing meat and fish (SFW) and heat treatment (60 °C - 10 min) applied on supermarket food waste containing meat and fish (SFW + MF).
